# Does Marriage Make Us Healthier? Inter-Country Comparative Evidence from China, Japan, and Korea

**DOI:** 10.1371/journal.pone.0148990

**Published:** 2016-02-10

**Authors:** Rong Fu, Haruko Noguchi

**Affiliations:** 1 Graduate School of Economics, Waseda University, Tokyo, Japan; 2 Faculty of Medicine, University of Tsukuba, Ibaraki, Japan; 3 Faculty of Political Science and Economics, Waseda University, Tokyo, Japan; University of Rochester, UNITED STATES

## Abstract

**Objectives:**

This study focuses on East Asian countries and investigates the difference in the marriage premium on the health-marriage protection effect (MPE) between younger and older generations and the intra-couple education concordance effect (ECE) on the health of married individuals. This study used inter-country comparative data from China, Japan, and Korea.

**Methods:**

This study focused on individuals (n = 7,938) in China, Japan, and Korea who were sampled from the 2010 East Asian Social Survey. To investigate MPE and ECE, four health indicators were utilized: a physical and mental components summary (PCS and MCS), self-rated health status (*D*_*self*_), and happiness level (*D*_*happy*_). Ordinary least squares regression was conducted by country- and gender-specific subsamples.

**Results:**

We found that the MPE on PCS, MCS, and *D*_*self*_ was more significant for the older generation than for the younger generation in both China and Japan, whereas the results were inconclusive in Korea. With regard to the ECE on happiness (*D*_*happy*_), for both men and women, couples tend to be happier when both the husband and the wife are well educated (“higher balanced marriage”) compared to couples with a lower level of educational achievement (“lower balanced marriage”). Significant benefits from a “higher balanced marriage” on MCS and *D*_*self*_ were observed for women only. In contrast, no statistically significant differences in health status were observed between “higher balanced marriage” couples and couples with different levels of educational achievements (“upward marriage” or “downward marriage”).

**Conclusions:**

This study found that (1) the MPE was more significant for the older generation, and (2) the health gap, particularly the happiness gap, between higher- and lower-balanced married couples was significant. The inter-country comparative findings are useful to explain how the role of marriage (and therefore of family) on health has been diluted due to the progress of industrialization and modernization.

## Introduction

Numerous studies have found that health is significantly associated with various socioeconomic and demographic factors, including marriage. Previous findings support the positive impact of marriage on health [1–4], known as the marriage protection effect (MPE). Married persons are found to be more likely to enjoy better physical health, such as lower mortality and/or morbidity [4,5], and better mental health than unmarried persons [6]. There are three reasons why further analyses of this issue are necessary.

First, although numerous works have demonstrated the MPE using various health indicators, to the best of our knowledge, few of these studies have investigated differences in MPE between a pair of different *age cohorts*. Furthermore, little evidence is based on nationally representative datasets. In this study, we choose age 50 as the criterion to distinguish between people of the older generation (50+) and the younger generation (50-), and then we investigate whether the MPE differs between the two generations. We do not use the age of 65, which is generally used in other studies, as a criterion because we exclude respondents who are older than 65 (65+) from our analysis to avoid unnecessary heterogeneity bias. The characteristics of respondents who are 65+ differ significantly from those who are younger than 65 in the data used in this study. For example, the rate of illiteracy or elementary school as the highest educational level for individuals 65 and older is 36.72%, whereas this rate is 17.33% for those younger than 65.

There are two plausible explanations for different MPE across generations. One prevalent explanation is that different attitudes toward marriage across generations lead to various MPEs [7–9]. Generation-specific attitudes toward marriage may be the result of changing social values with regard to gender roles and family formation [10]. These changes were remarkable in East Asian countries in the late 20th century. Another explanation is that the role of marriage (and therefore of family) on the health of household members has been diminishing over generations because public health care and the social security system have gradually been developed and have taken over the role of the family. Therefore, it is worthwhile to investigate the MPE of younger and older generations separately and to evaluate the MPE gap between these two cohorts.

Second, there is little evidence to indicate whether and how intra-couple similarity in socioeconomic characteristics affects health status. Some studies have focused on intra-couple correlations for physical characteristics, such as weight, height, and frame size. Dufouil and the EVA Study Group explored the influence of intra-couple similarity on psychological state [11], and Schafer and Keith found that the weights of the individuals in retired couples were significantly correlated, which might reflect the shared lifestyle of old people [12]. In this work, rather than examining the intra-couple correlation of physical characteristics, we are interested in investigating how concordance or disparity of educational achievement between husbands and wives influences their health status–the education concordance effect (ECE). Education, which is a primary and comprehensive indicator of socioeconomic status, particularly social rank, may be positively associated with health. Higher educational attainment not only indicates a greater ability to acquire, assimilate, and analyze health-related information, which may lead people to more appropriate health behavior and a better lifestyle [13], but also is associated with better psychological and socioeconomic status, such as higher self-esteem, stronger social networks, and higher family income [13], which, in turn, may improve health significantly [3]. Furthermore, intra-couple concordance/disparity of educational achievement may reflect the degree of both mental and physical interactions within couples, which affect their health status (e.g., better mental health because of similar values within couples or better physical health because of appropriate suggestions regarding health behavior by a spouse). By evaluating changes in health status in various combinations of educational achievement within couples, we examine whether well-matched marriages—from the perspective of education—benefit people’s health more than unmatched marriages do.

Finally, the impacts of marriage on people’s life vary diametrically among regions where socio-cultural backgrounds are dissimilar to each other. Nevertheless, most previous studies have evaluated the MPE in Western countries, such as the United States and Canada. Hence, this study will focus on East Asian countries. Because these countries have completely different socio-cultural characteristics from Western countries, our results regarding the MPE might differ from previous studies. Recent research on the MPE in East Asia by Chung and Kim shows a strong and positive association between marriage satisfaction and self-rated health status (SRH) in China, Taiwan, and Korea, but this is not the case for Japan [14]. Specifically, married people with a higher level of marriage satisfaction are more likely to claim that their SRH is “excellent” or “very good” compared with unmarried people. Several aspects distinguish our study from the study by Chung and Kim. First, we used the East Asia Social Survey 2010, which is the most recent available data that are appropriate for our research purposes. Chung and Kim used the 2006 version of the same survey, which had the theme “Family in East Asia”. The 2010 version of this dataset focused on “Health and Society in East Asia” and was designed to measure the health status of respondents; thus, we evaluate marriage effects in terms of more comprehensive indicators of health, such as the physical component summary (PCS) and mental component summary (MCS), as well as SRH as used in Chung and Kim. More importantly, our scope differs from that of Chung and Kim. They focused on the association between marriage satisfaction and health, whereas we investigate the MPE—health gap between married and unmarried people as well as the ECE within married couples.

We focus on three East Asian countries, China, Japan, and Korea, to investigate the MPE for two age cohorts (under 50 and 50+) and to evaluate the gap in the MPE between these cohorts [15,16]. Furthermore, we extract only the married population to examine ECE and consider how their health status varies with intra-couple similarity in educational achievement. We test three hypotheses as follows. (1) The MPE is more significant for the older generation (50+) than for the younger generation (under 50) [**Hypothesis on MPE**]. (2) A person may enjoy better health if intra-couple concordance of educational achievement is attained (that is, if both the husband and the wife are well educated) compared to couples with lower educational achievement, and vice versa [**Hypothesis on ECE Type I**]. (3) A person may enjoy better health if intra-couple concordance of educational achievement is attained compared to couples with different levels of educational achievement (that is, either the husband or the wife is well educated, and vice versa) [**Hypothesis on ECE Type II**].

The outline of this paper is as follows. The next section describes our dataset and the measurements. The third section introduces our empirical strategies, followed by the fourth section of statistical results. The fifth section provides a discussion of the results, and the final section concludes.

## Methods

### Data

This study uses the East Asia Social Survey, a unique inter-country integrated dataset that is a combination of the Chinese General Social Survey (CGSS), the Japanese General Social Survey (JGSS), the Korean General Social Survey (KGSS), and the Taiwan Social Change Survey (TSCS). In each country, the survey was conducted by a corresponding institution in 2006, 2008, 2010, and 2012, and a specific issue was the focus of each year: “Family” in 2006, “Culture and Globalization” in 2008, “Health and Society” in 2010, and “Social Network” in 2012.

The EASS data archive provides publicly available data from respondents whose identities are undisclosed. Ethical approval for this study was granted by the Research Ethical Review Committee of Waseda University, Tokyo, Japan.

Because this study focused on “Health and Society” in East Asian countries, we used the 2010 version, which surveyed various health indicators as well as demographic and socioeconomic factors. Furthermore, we used data on China, Japan, and Korea because two significant health indicators—the physical component summary (PCS) and the mental component summary (MCS)–are not available in the data for Taiwan. Respondents in the survey were Chinese aged 18 and older (18+), people living in Japan aged 20–90, and people living in Korea aged 18+. The participants were randomly selected in each country. The survey was based on interviewing with the exception of some variables answered by a self-administered method in Japan. The numbers of participants in the study populations were 5,370, 4,500, and 2,500, and the number and rate of valid responses were 3,866 (71.9%), 2,496 (62.1%), and 1,576 (63%) in China, Japan, and Korea, respectively. As mentioned above, we narrowed our respondents to those who were under 65 (65-) to avoid cohort bias from those who were 65+.

### Measurements

Next, we define marriage and health status. For marriage, we define the dichotomous variable *married* as one if a respondent is married or cohabiting. For health status, four indicators are utilized to measure both physical and mental health in Table 1.

**Table 1:** Indicators of Health Status.

Indicators (Variable Name)	Definition
Physical Component Summary (PCS)	A physical distinct, higher-order summary score aggregated from eight SF-12 subscales: General Health, Role Physics, Physical Functioning, Bodily Pain, Mental Health, Role Emotion, Social Functioning, Vitality
Mental Component Summary (MCS)	A mental distinct, higher-order summary score aggregated from eight SF-12 subscales (as above)
Self-rated Health (*D*_*self*_)	*D*_*self*_ = 1 if self-rated health is “excellent" or “very good", 0 otherwise; where self-rated health is categorized in 5 ranks as follows: 1 as “excellent”, 2 as “very good”, 3 as “good”, 4 as “fair”, and 5 as “poor”
Happiness Level (*D*_*happy*_)	*D*_*happy*_ = 1 if happiness level is “very happy” or “happy”, 0 otherwise; where happiness level is categorized in 5 ranks as follows: 1 as “very happy”, 2 as “happy”, 3 as “either”, 4 as “unhappy”, 5 as “very unhappy”

The first two indicators are the PCS and MCS, which are aggregated from eight subscales in the Short Form 12-item (version 2) survey. Each subscale included 0 to 100 points, and we conducted factor analysis to extract two main factors, PCS and MCS. The other two indicators were dummy variables; self-rated health (*D*_*self*_) took the value of one if a respondent self-assessed his/her health status as “Excellent” or “Very Good”; similarly, happiness level (*D*_*happy*_) had a value of one if a respondent reported being “Very Happy” or “Happy”. Basic statistics are shown in Table 2.

**Table 2:** Basic Statistics^a^.

Variable Name	China (n = 3277)		Japan (n = 1721)		Korea (n = 1312)	
	mean	sd	mean	sd	mean	sd
PCS	49.31	(10.71)	51.79	(7.64)	49.34	(10.61)
MCS	52.00	(9.43)	47.06	(9.52)	48.90	(10.86)
*D*_*self*_	0.62	(0.49)	0.21	(0.41)	0.57	(0.50)
*D*_*happy*_	0.70	(0.46)	0.59	(0.49)	0.48	(0.50)
Married	0.83	(0.38)	0.73	(0.44)	0.66	(0.48)
Age	42.71	(12.13)	45.00	(12.41)	39.76	(11.77)
Male	0.48	(0.50)	0.44	(0.50)	0.48	(0.50)
High School	0.36	(0.48)	0.94	(0.23)	0.86	(0.34)
College	0.17	(0.38)	0.45	(0.49)	0.54	(0.49)
Income above average	0.09	(0.29)	0.16	(0.36)	0.20	(0.40)

^a^ Individuals older than 65 (65+) are excluded to avoid the cohort bias.

With regard to health indicators, three out of four were highest in China, but the PCS was highest in Japan. For the demographic characteristics, the rate of being *married* was highest in China (83%), and the average *age* was highest in Japan (45). Education level was polarized across countries. The rates of respondents who graduated from high school or above were 94% and 86% in Japan and Korea, respectively, but this rate was only 36% in China. Regarding college education, the gaps between China and the other two countries narrowed; nevertheless, only 17% of Chinese respondents graduated from college, whereas this rate was 45% and 54% for Japanese and Koreans, respectively. For income level, the rate of those who believed their family income was above average was relatively higher in Korea (20%) than in China (9%) and Japan (16%).

## Empirical Strategies

First, we focus on the difference in health status between married and unmarried respondents to verify the MPE [**Hypothesis on MPE**]. Second, we evaluate the effect of intra-couple similarity with respect to educational achievement on a couple’s health status [**Hypotheses on ECE Type I and Type II**].

### Hypothesis on MPE

Our regression formula is quite straightforward, as follows:

$$H_{i}=\alpha_{0}+\alpha_{1}married+\;\alpha_{2}age_{50}+\;\alpha_{3}married\times age_{50}+\;\sum \alpha_{i}x_{i}+u_{i},$$ (1)

where *H*_*i*_ corresponds to the four health indicators defined in Table 1. Our concerns are the coefficients on *married*, *age*_50_, and the interaction term *married* × *age*_50_. *α*_1_ is expected to be positive to confirm the MPE for respondents 50-; *α*_2_ is non-positive for health deterioration due to aging; and *α*_3_ is positive because the MPE is expected to be stronger for those in the older cohort. We also derive *α*_1 +_ *α*_3_ by the delta method to confirm the MPE for respondents 50+. Moreover, *x*_*i*_ are covariates including gender, years of education, self-rated family income level, self-placement of social class, whether the respondent is the main caregiver within the family, and whether the respondent worried about health care accessibility and cost.

### Hypotheses on ECE Type I and Type II

First, we define the concordance of educational achievement between couples—whether both husbands and wives are well educated. Because of the polarized educational levels, a single criterion for “well educated” could not be determined across countries. Therefore, we define a higher education level (H) to be high school graduation or above in China and college graduation or above in Japan and Korea. Correspondingly, we define a lower education level (L) for Chinese respondents to be junior high school graduation or below and for Japanese and Korean respondents to be high school graduation or below. As a result, the rates of “well-educated” respondents are more balanced across countries: 36% in China, 45% in Japan, and 54% in Korea.

According to the definition of high and low education levels, we categorize respondents into four groups: “LH”, “HL”, “LL”, and “HH”. “LH” indicates a respondent who is categorized into “L” but whose spouse has achieved “H”, which is denoted as “upward marriage” (from the perspective of the respondent). Correspondingly, we denote “HL” as “downward marriage”, “LL” as “lower balanced marriage”, and “HH” as “higher balanced marriage”.

Our primary concern is the health gap between “HH” and “LL” (ECE type I) and between “HH” and “HL” / “LH” (ECE type II). Thus, our regression formula is as follows:

$$H_{i}=\beta_{0}+\beta_{1}LL+\;\beta_{2}LH+\;\beta_{3}HL+\;\sum \beta_{i}y_{i}+\epsilon_{i},$$ (2)

where *β*_0_ represents the average health of respondents in “HH”, and *β*_1_ controls the health gap between “LL” and “HH”, which is expected to be negative. Similarly, *β*_2_(*β*_3_)is expected to be negative, which would measure the health gaps between “LH” (“HL”) and “HH”. *y*_*i*_ stands for covariates including the ages of the respondent and the respondent’s spouse, years of education, self-rated family income level, self-placement of social class, whether the respondent is the main caregiver within the family, and whether the respondent is worried about health care accessibility and cost.

To make the MPE comparable among health indicators, we take a logarithm of PCS and MCS so that all coefficients indicate the percentage change (%) in the health indicators. For the robustness check, we also implement OLS for the non-logarithm value of PCS and MCS and a logistic regression for *D*_*self*_ and *D*_*happy*_. However, the results are almost the same. All regressions are run separately for men and women and performed using Stata 14.0.

## Results

### Marriage Protection Effect

Before we discuss the regression results, we will briefly examine the age structure and marriage ratio of our samples by age groups across countries.

**Fig 1:**
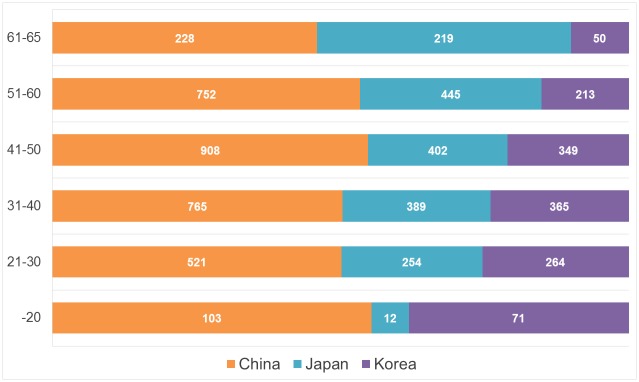
Comparative Age Structures in China, Japan, and Korea. 10-year age groups in which “younger than 20 (-20)” in Japan includes only those who are 20. Numbers in bars indicate corresponding numbers of observation.

**Fig 2:**
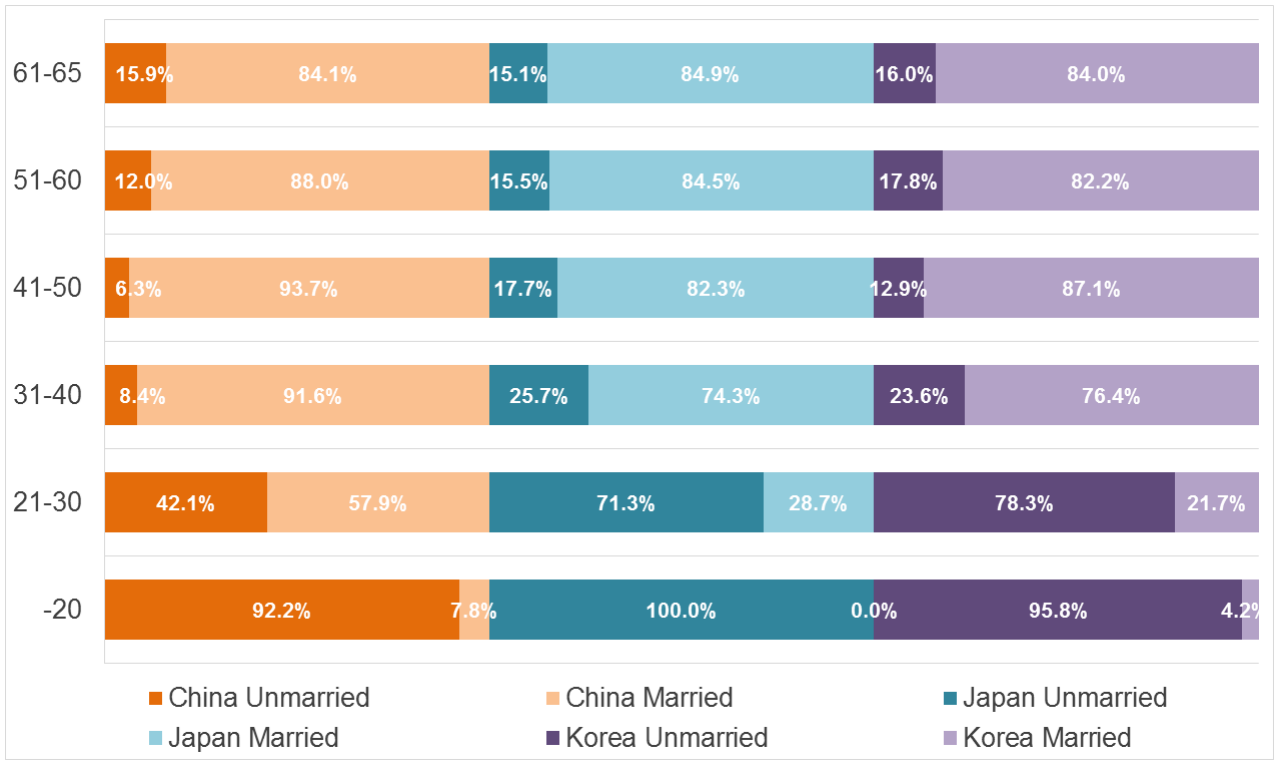
Marriage by Age Groups in China, Japan, and Korea. 10-year age groups in which “younger than 20 (-20)” in Japan includes only those who are 20. Numbers in bars indicate percentage of married/unmarried individuals in each country.

We construct the 10-year age group from the “younger than 20 (-20)” through “61 and older and 65 and younger (61–65)” groups. Note that the respondents in Japan were aged 20–90, so the youngest age group in Japan includes only those who are 20 years old. Fig 1 shows that the rate of Chinese respondents across age groups is relatively more stable than the Japanese and Korean ones, which vary remarkably across age groups. Specifically, the rate of Japanese respondents increases notably with age, whereas Korean respondents show an opposite trend in which the rate decreases with age. With regard to the marriage ratio shown in Fig 2, all countries show a similar trend such that the marriage ratio increases according to age. The marriage ratios are doubled in the 30s compared with the rate in the 20s in all countries (from 57.9% to 91.6% in China; from 28.7% to 74.3% in Japan; and from 21.7% to 76.4% in Korea). Although the trend seems to be the same, the marriage ratio in the 20s differs between China (more than 50%) and Japan and Korea (only approximately 20%-30%). This finding indicates that Japanese and Koreans may be postponing marriage.

Table 3 shows the results of MPE, shown by Eq 1. *Married (50-)* corresponds to *α*_1_ in Eq 1, *MPE Gap* to *α*_3_, *Married (50+)* to *α*_1_ + *α*_3_ derived by the delta method, and *Cons*. is the average health of unmarried people who are 50-.

**Table 3:** Marriage Protection Effect by Generation^a^.

	China (n = 3156)			Japan (n = 1670)			Korea (n = 1257)		
	Coef.^b^	p-value^c^	t/z-value^c^	Coef.	p-value^c^	t/z-value	Coef.	p-value^c^	t/z-value
**PCS** ^d^									
*Married (50-)*	0.019	(0.12)	(1.56)	0.005	(0.65)	(0.45)	-0.030 **	(0.02)	(-2.26)
*Married (50+)*	0.096 ***	(0.01)	(2.61)	0.018	(0.35)	(0.94)	0.207 ***	(0.01)	(2.65)
*MPE Gap*	0.077 **	(0.05)	(1.98)	0.013	(0.55)	(0.59)	0.238 ***	(0.00)	(2.99)
*Cons*.	3.765 ***	(0.00)	(157.53)	3.800 ***	(0.00)	(106.06)	3.741 ***	(0.00)	(84.66)
**MCS** ^d^									
*Married (50-)*	0.008	(0.42)	(0.81)	0.048 ***	(0.00)	(2.90)	0.028 *	(0.07)	(1.84)
*Married (50+)*	0.072 ***	(0.00)	(3.28)	0.051 *	(0.06)	(1.86)	0.059	(0.27)	(1.11)
*MPE Gap*	0.064 ***	(0.01)	(2.63)	0.003	(0.93)	(0.08)	0.031	(0.58)	(0.56)
*Cons*.	3.838 ***	(0.00)	(216.38)	3.637 ***	(0.00)	(77.04)	3.743 ***	(0.00)	(88.19)
***D***_***self***_									
*Married (50-)*	-0.029	(0.21)	(-1.26)	-0.029	(0.35)	(-0.91)	-0.104 ***	(0.00)	(-3.30)
*Married (50+)*	0.074 *	(0.08)	(1.75)	0.050 *	(0.07)	(1.77)	0.026	(0.72)	(0.36)
*MPE Gap*	0.103 **	(0.03)	(2.13)	0.079 **	(0.04)	(1.89)	0.131	(0.10)	(1.64)
*Cons*.	0.571 ***	(0.00)	(14.20)	0.125	(0.14)	(1.46)	0.410 ***	(0.00)	(5.04)
***D***_***happy***_									
*Married (50-)*	0.092 ***	(0.00)	(3.77)	0.233 ***	(0.00)	(7.30)	0.136 ***	(0.00)	(4.17)
*Married (50+)*	0.133 ***	(0.00)	(2.95)	0.249 ***	(0.00)	(5.43)	-0.035	(0.65)	(-0.46)
*MPE Gap*	0.041	(0.43)	(0.79)	0.017	(0.76)	(0.30)	-0.171 **	(0.04)	(-2.06)
*Cons*.	0.362 ***	(0.00)	(8.61)	0.064	(0.49)	(0.69)	0.026	(0.76)	(0.31)

^a^ The model was adjusted for gender, education, family income level, social class, whether the respondent was the main caregiver within the family, and whether the respondent worried about health care accessibility and cost.

^b^ * p<0.1 ** p<0.05 *** p<0.01

^c^ p-value, Heteroscedasticity-robust t- and z-statistics in parentheses

^d^ Logarithm of PCS, Physical Component Summary; MCS, Mental Component Summary

The coefficients on *Married (50-)* do not show any conclusive trends across countries or health indicators. Only the coefficients of *D*_*happy*_ are almost always significantly positive in all countries (9.2% in China, 23.3% in Japan, 13.6% in Korea), implying better happiness levels for married people under 50. Otherwise, no consistent results are found.

In contrast, with regard to coefficients on *Married (50+)*, the results differ by country. In China, the MPE for the older cohort (50+) is confirmed by significantly positive coefficients on all health indicators (9.6% on PCS, 7.2% on MCS, 7.4% on *D*_*self*_, and 13.3% on *D*_*happy*_). In Japan, the MPE for the 50+ cohort is also generally confirmed except on PCS (5.1% on MCS, 5.0% on *D*_*self*_, and 24.9% on *D*_*happy*_), whereas the MPE is confirmed only on PCS (20.7%) in Korea.

We then test our hypothesis on MPE by the coefficients on *MPE Gap*. The hypothesis seems to be supported in China and Japan (although weakly in Japan), but it seems to be inconclusive in Korea. In China, the MPE gaps are significantly positive except on *D*_*happy*_. For instance, the MPE gap on PCS is 7.7%, indicating that the MPE on PCS for married Chinese in the older cohort (50+) is higher by 7.7 percentage points than the one in the younger cohort (50-). Similarly, the MPE on *D*_*self*_ for married Japanese 50+ tends to be higher by 7.9 percentage points than the one for the 50- group. However, in Korea, although the coefficient of *MPE Gap* on PCS is significantly positive (23.8%), the one on *D*_*happy*_ is significantly negative (-17.1%).

**Fig 3:**
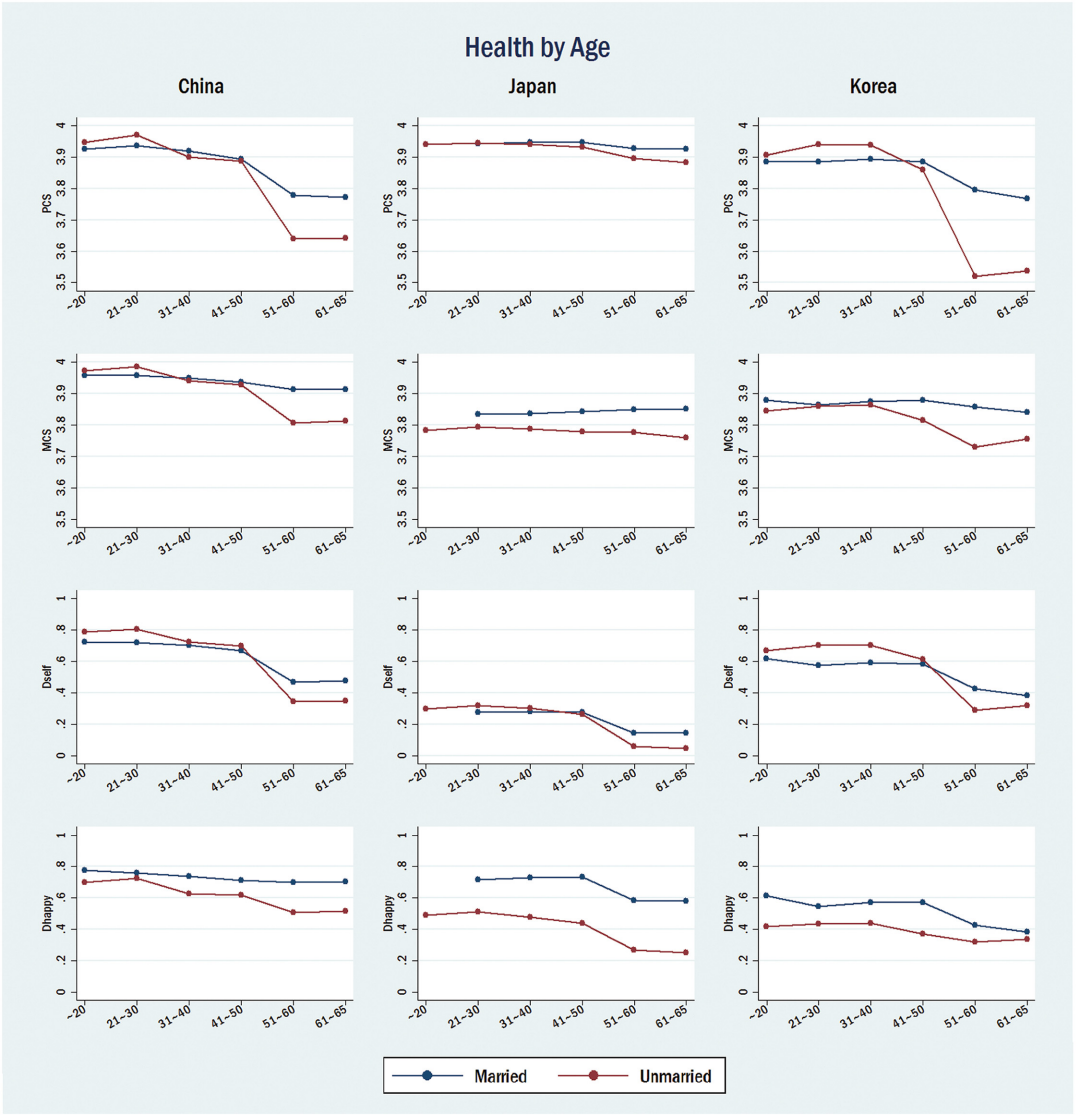
Predicted Marriage Protection Effects with Age. Rows correspond to health indicators, columns to countries. Blue line denotes health of married respondents, and red line denotes health for unmarried respondents.

Our findings are also confirmed in Fig 3, which illustrates predicted health by age groups. The blue line denotes the health of married respondents, and the red line denotes the health of unmarried respondents. With regard to PCS, MCS, and *D*_*self*_, the MPE gaps increase suddenly after age 50 in China; the same pattern is found, albeit slightly, only on *D*_*self*_ in Japan, whereas a notably enlarged gap is found only on PCS in Korea. For the MPE on *D*_*happy*_, the gap between married and unmarried people is quite stable before and after age 50 in China and Japan, whereas the gap converges in Korea after age 50. Regarding the coefficients on *D*_*happy*_ in Table 3, we find an insignificant *MPE gap* on *D*_*happy*_ in China and Japan and a negative gap in Korea, most likely due to the strong MPEs for the 50- group. In other words, marriage may make people feel happier regardless of generation.

### Education Concordance Effect

Before we explain the regression results, we briefly review the intra-couple concordance of educational achievement by gender in each country.

**Fig 4:**
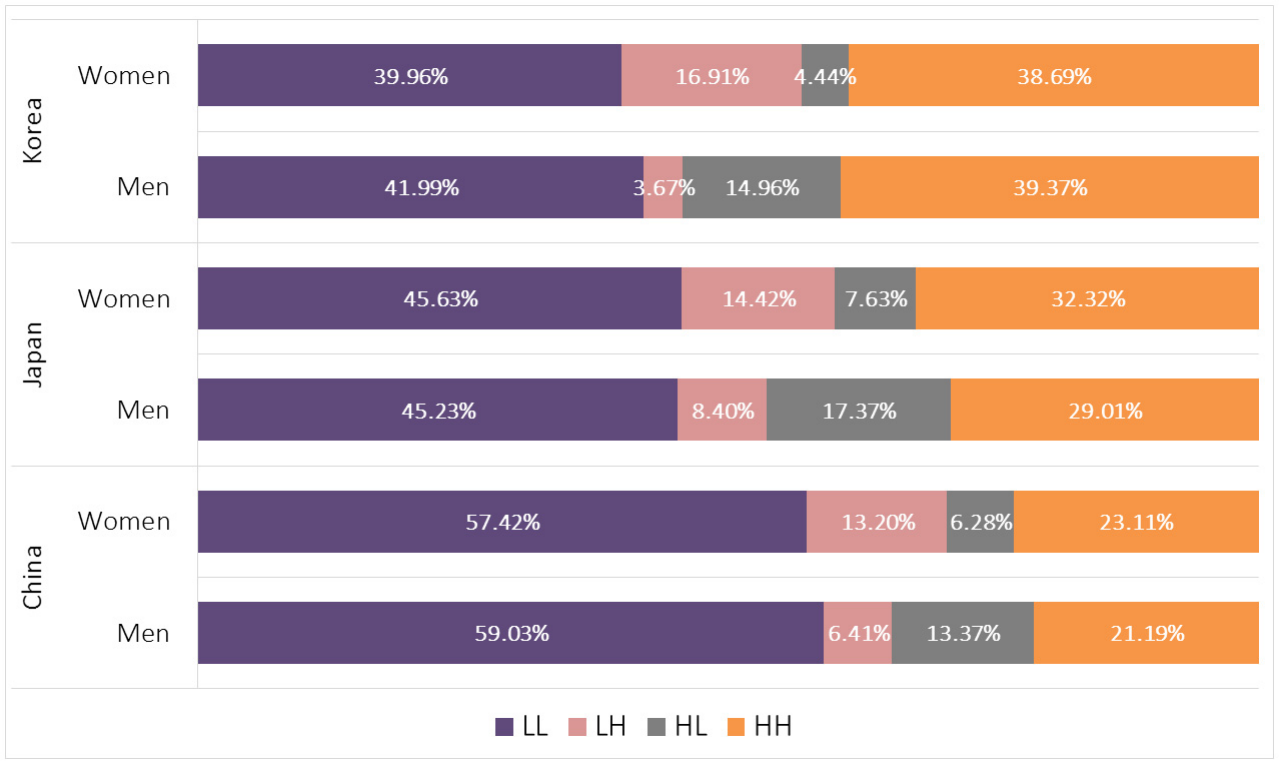
Education Concordances by Gender in China, Japan, and Korea. Percentages in bars indicate corresponding ratios of intra-couple concordance of educational achievement by gender in each country.

Fig 4 shows that the rates of “LL” and “HH” are almost the same for men and women (e.g., in China, “LL” is 59.03% and 57.42% for men and women, respectively). However, the rates differ across countries. The rate of “LL” is relatively high in China compared to Japan and Korea, whereas the rate of “HH” is highest in Korea of the three countries. Regarding “LH” and “HL”, the rates vary by gender. Regardless of country, women tend to prefer “upward marriage” (“LH”) compared with “downward marriage” (“HL”), whereas men make the opposite decision regarding marriage.

Table 4 shows the regression results of ECE for both men and women. “LL”, “LH” and “HL” represent *β*_1_, *β*_2_, *β*_3_ in Eq 2, and *Cons*. gives the average health of respondents in a group of “HH” as a benchmark. *β*_1_ (coefficients on “LL”) shows the result of testing our hypothesis on ECE type I such that respondents in “HH” may enjoy better health than those in “LL”. *β*_2_ and *β*_3_ (coefficients on “LH” and “HL”) test our hypothesis on ECE type II such that respondents in “HH” have better health than those in “LH” or “HL”.

**Table 4:** Education Concordance Effect by Gender and Country^a^.

Gender	Health Indicators	Intra-couple Education Concordance	China (n = 1229(M) n = 1349(W))			Japan (n = 510(M) n = 703(W))			Korea (n = 366(M) n = 449(W))		
			Coef.^b^	p-value^c^	t-value^c^	Coef.	p-value^c^	t-value	Coef.	p-value^c^	t-value
**MEN**	**PCS** ^d^	HL	0.006	(0.72)	(0.36)	-0.042 **	(0.04)	(-2.08)	0.009	(0.71)	(0.38)
		LH	0.020	(0.36)	(0.92)	0.012	(0.67)	(0.43)	-0.059	(0.19)	(-1.32)
		LL	-0.038 ***	(0.01)	(-2.73)	-0.027	(0.10)	(-1.63)	-0.064 **	(0.02)	(-2.31)
		Cons	4.177 ***	(0.00)	(133.34)	3.956 ***	(0.00)	(81.38)	4.040 ***	(0.00)	(72.35)
	**MCS** ^d^	HL	-0.021	(0.23)	(-1.19)	-0.033	(0.26)	z(-1.14)	0.041	(0.18)	(1.33)
		LH	0.000	(0.99)	(-0.02)	0.001	(0.97)	(0.04)	-0.062	(0.46)	(-0.75)
		LL	-0.019	(0.11)	(-1.62)	-0.023	(0.33)	(-0.97)	-0.006	(0.81)	(-0.23)
		Cons	4.021 ***	(0.00)	(148.76)	3.864 ***	(0.00)	(65.55)	3.888 ***	(0.00)	(62.72)
	***D***_***self***_	HL	0.072 *	(0.10)	(1.67)	-0.073	(0.19)	(-1.32)	0.118 *	(0.08)	(1.74)
		LH	0.041	(0.47)	(0.72)	0.010	(0.90)	(0.13)	-0.047	(0.74)	(-0.33)
		LL	-0.013	(0.69)	(-0.40)	0.068	(0.18)	(1.35)	-0.041	(0.54)	(-0.61)
		Cons	1.069 ***	(0.00)	(15.96)	0.589 ***	(0.00)	(4.43)	0.790 ***	(0.00)	(5.65)
	***D***_***happy***_	HL	-0.087 **	(0.04)	(-2.05)	-0.147 **	(0.02)	(-2.36)	0.037	(0.61)	(0.51)
		LH	0.029	(0.56)	(0.58)	-0.043	(0.53)	(-0.63)	0.122	(0.30)	(1.03)
		LL	-0.083 ***	(0.00)	(-2.86)	-0.102 **	(0.03)	(-2.17)	-0.117 *	(0.07)	(-1.80)
		Cons	0.783 ***	(0.00)	(11.89)	1.198 ***	(0.00)	(10.59)	0.602 ***	(0.00)	(4.21)
**WOMEN**	**PCS** ^d^	HL	0.021	(0.40)	(0.84)	0.013	(0.51)	(0.66)	-0.069	(0.30)	(-1.04)
		LH	-0.011	(0.62)	(-0.50)	0.006	(0.72)	(0.36)	-0.008	(0.82)	(-0.23)
		LL	-0.055 ***	(0.00)	(-3.92)	-0.039 **	(0.01)	(-2.56)	-0.007	(0.80)	(-0.26)
		Cons	4.132 ***	(0.00)	(121.44)	3.973 ***	(0.00)	(105.26)	4.086 ***	(0.00)	(53.34)
	**MCS** ^d^	HL	0.017	(0.41)	(0.82)	0.007	(0.84)	(0.20)	-0.009	(0.88)	(-0.15)
		LH	-0.021	(0.29)	(-1.06)	-0.007	(0.79)	(-0.27)	0.041	(0.25)	(1.16)
		LL	-0.042 ***	(0.00)	(-3.35)	-0.042 **	(0.04)	(-2.02)	0.020	(0.50)	(0.68)
		Cons	3.983 ***	(0.00)	(136.93)	3.732 ***	(0.00)	(66.25)	3.819 ***	(0.00)	(61.86)
	***D***_***self***_	HL	0.084	(0.10)	(1.63)	-0.073	(0.25)	(-1.15)	-0.057	(0.62)	(-0.50)
		LH	0.035	(0.42)	(0.81)	-0.084 *	(0.10)	(-1.67)	-0.050	(0.47)	(-0.73)
		LL	-0.020	(0.53)	(-0.63)	-0.047	(0.24)	(-1.17)	-0.115 *	(0.07)	(-1.84)
		Cons	1.031 ***	(0.00)	(15.03)	0.598 ***	(0.00)	(5.89)	0.871 ***	(0.00)	(6.54)
	***D***_***happy***_	HL	-0.012	(0.78)	(-0.27)	0.041	(0.56)	(0.59)	-0.053	(0.67)	(-0.43)
		LH	-0.049	(0.20)	(-1.28)	-0.052	(0.34)	(-0.95)	-0.044	(0.53)	(-0.63)
		LL	-0.137 ***	(0.00)	(-5.08)	-0.087 **	(0.03)	(-2.12)	-0.036	(0.56)	(-0.58)
		Cons	0.710 ***	(0.00)	(11.64)	0.846 ***	(0.00)	(8.01)	0.519 ***	(0.00)	(3.89)

^a^ The model was adjusted for age, spouse’s age, education, family income level, social class, whether the respondent was the main caregiver within the family, and whether the respondent worried about health care accessibility and cost.

^b^ * p<0.1 ** p<0.05 *** p<0.01

^c^ p-value, Heteroscedasticity-robust t statistic in parentheses

^d^ Logarithm of PCS, Physical Component Summary; MCS, Mental Component Summary

For the hypothesis on ECE type I, we find powerful evidence on *D*_*happy*_. Except for Korean women, significantly negative coefficients of “LL” are found. The largest gap is for Chinese women in “LL”, whose happiness level declined by 13.7% compared with those in “HH”. Considering ECE type I by gender and country, the effects of “LL” for men become statistically significant on PCS and *D*_*happy*_ in China and Korea, but this is true only for *D*_*happy*_ in Japan. For women, the coefficients of “LL” are statistically significant on PCS, MCS, and *D*_*happy*_ in China and Japan but only for *D*_*happy*_ in Korea. Thus, we conclude that women are more likely to be influenced by type I ECE than men are and that type I ECE may benefit Chinese respondents’ health the most among three countries.

We could not find conclusive evidence for ECE type II. None of the coefficients of upward marriage (“LH”) are statistically significant for both genders, except for Japanese women’s *D*_*self*._ Japanese women in “LH” are less likely to declare themselves “Very Happy” or “Happy” by 8.4 percentage points than those in “HH”. For downward marriage (“HL”), no statistically significant effects are found for women regardless of country. However, “HL” has a significant positive impact on *D*_*self*_ and *D*_*happy*_ of Chinese and Korean men. Specifically, these groups are 7.2% more likely in China and 11.8% more likely in Korea to report better health status (*D*_*self*_) in the case of “downward marriage”, although Chinese men tend to report lower *D*_*happy*_ by 8.7 percentage points. In general, “upward marriage” has no influence on health; however, “downward marriage” might affect health for men only. However, the evidence remains insufficient.

## Discussion

Using nationally representative data in China, Japan, and Korea, we investigated the hypotheses on MPE and ECE (type I and II). Our results support our first hypothesis such that the MPE is *more significant* for people 50+ than for those 50-, at least in China and Japan (but weakly in Japan). With regard to ECE, ECE type I on D_*happy*_ is confirmed, and the impacts are more significant for women than for men. However, no conclusive evidence could be found for ECE type II.

The *more significant* MPE for the older generation may reflect different stages of industrialization, socioeconomic development, and demographic transitions from one generation to another in each country. Due to the progress of industrialization and modernization, the importance of marriage (and therefore of family) on health has been diluted because many substitutional entities, such as education, public health care (including medical and long-term care), and the social security system have gradually taken over the role of marriage for health protection. Therefore, the MPE is more significant for the older generation. In their era, marriage played an important role as a shelter for people’s health, both physical and mental.

This argument is supported not only by the positive coefficients on *MPE Gap* (the difference in the MPE between older and younger generations) within China and Japan but also by the different number of significant coefficients on *MPE Gap* between the two countries. Specifically, the significant *MPE Gap* is confirmed in Japan only for *D*_*self*_, whereas it is confirmed in China for PCS, MCS and *D*_*self*_. This is most likely because the protection of marriage differs for Japanese 50+ and Chinese 50+, although they are both categorized as the old generation. Assuming that people marry at age 25 on average, then a representative Japanese person in his or her 60s in 2010 (mean age of 50+ age cohort) was more likely be married in the 1970s, when Japan was undergoing incomparably rapid economic development, which is almost the same as China has been experiencing in the 2000s. Moreover, the Japanese marriage squeeze ratio, indicating the ratio of marriageable men to three-year-younger women (e.g., the ratio of men aged 25–29 over women aged 22–26), was approximately 1.21 in 1975 [17], which is identical to the ratio in China in 2000 [18]. This finding implies that the marriage market situation and the attitude toward marriage in Japanese society in the 1970s might be similar to the situation in China in the 2000s, which could lead to the similarity of the MPE. Therefore, the MPE of Japanese 50+ corresponds to that of Chinese in their 40s or younger, which explains the weaker *MPE Gap* in Japan than in China.

Nevertheless, Korea distinguishes from China and Japan with the inconclusive *MPE Gap*. The more significant MPE on PCS is found for Korean 50+, whereas the situation becomes reversed for *D*_*happy*_. One explanation is the de- and re-Confucianization processes along with the drastic development during the second half of the 20th century in Korea, which is unique in East Asia [19]. In the early stage of industrial capitalism process, Korea regarded traditional Confucian family-centered social order as an obstacle of economy development that shall be swept out, which resulted in diminishing importance of family and marriage in Korean society. In the rapid modernization during 1970s and early 1980s, however, Korea began to make great efforts to reanimate the Confucianism for two purposes: to rediscover and redefine its national ideology, and to compete with and distinguish from other East Asian countries (particular Japan) that were also in process of Westernization [19,20]. As a result, familism (i.e. marriage and family) again became important in all aspects of daily life in Korea [20].

Similar to China and Japan, industrialization in Korea dilutes the importance of marriage on health protection across generations (i.e. stronger MPE on PCS for older Korean), yet recursion of Confucianization together with the industrialization may lead to contradictory attitudes toward marriage and family even within one generation, making evidence for hypotheses on MPE not definitive in Korea.

This study also supports the hypothesis on ECE type I for *D*_*happy*_. Regardless of country and gender, “lower balanced marriage” couples are less likely to feel happy in their daily lives. Happiness is highly correlated with people’s personality, self-esteem, and aspects of life [21], which are affected by education. Hence, better education could contribute to a happier life [22,23]. When both the husband and wife are well educated, their happiness level may be multiplicative rather than additive. The multiplicative happiness is from mental harmony, higher family income, and more appropriate health behavior. We also find that women are more likely to be influenced by type I ECE than men are. For example, Japanese women in “LL” tend to report the disadvantage of PCS, MCS and happiness level, whereas this disadvantage is found only for happiness among married men. This finding could be explained by a social support theory [24]. Married women are more likely to be supported both mentally and physically by social networks compared to men, who tend to focus on conjugal relations and to rely on their spouses exclusively. Little difference in marriage balance between “LL” and “HH” is found for men, but women suffer unfavorable social networks due to a “lower balanced marriage”.

No significant ECE type II for “upward married” men (“LH”) and “downward married” women (“HL”) was observed in all countries. This finding is inconsistent with the traditional“doing-gender” agreement in East Asian society. Many previous studies [25] have argued that “breadwinner husbands and housekeeper wives make life happier”, indicating that men suffer from “upward marriage” (disqualification as a breadwinner), whereas women feel the opposite way. For example, Zuo and Bian found that Chinese women were happier if their husbands were more highly educated than themselves because “upward marriage” implies being financially secure [26]. Despite these previous findings, this study suggests that gender equality in East Asian countries might become far more prevalent than expected. As women’s education level increases along with the socio-economic value of women as human capital, being the breadwinner is no longer the exclusive responsibility of men. Moreover, “downward marriage” for women might no longer be a “rare event” in society. For instance, among Chinese people between the ages of 25 and 40, the rate of well-educated women who graduated from high school or above was 14.8% in 2000, which doubled to 29.9% in 2010 [18]. Meanwhile, the rate of educated men increased from 21.2% to 31.3%. This decrease in the gender gap on education is clearer if we examine the rate of postgraduates, which increased moderately from 0.34% to 0.39% for men during 2000–2010 but increased notably from 0.16% to 0.4% for women [18]. Likewise, the gender gap in education can also be observed in Japan and Korea, which should make “downward marriage” for women and “upward marriage” for men common events in East Asian society without either subjective or objective barriers.

## Conclusion

In sum, we find that (1) the marriage protection effect (MPE) is *more significant* for people 50+ than 50-; (2) the health gap, particularly the happiness gap, between higher- and lower-balanced married couples is significant; and (3) “upward marriage” for women and “downward marriage” for men might no longer be rare events and may not have a statistically significant impact on health on average.

Two limitations should be noted. One is that due to the cross-sectional data, we could not follow up the change in health status for each individual. Therefore, the results might be statistically biased by unobserved individual fixed effects. Another important limitation is that the stronger MPE gap in this study might be overestimated because we could not control for marriage duration, which was not available in the survey. Therefore, further research is required to offer more profound interpretations.
